# Supplementary material for: Bacteriophage Rescue Therapy of a Vancomycin-Resistant Enterococcus faecium Infection in a One-Year-Old Child following a Third Liver Transplantation
Source: Viruses. 2021 Sep 7;13(9):1785. doi: 10.3390/v13091785 (PMC8472888; doi:10.3390/v13091785)
Supplement: Supplementary file 1 [file viruses-13-01785-s001.zip › viruses-1341322-supplementary.pdf]

Supplementary Figures

Bacterial growth of target bacteria VRE*fm* untreated or in combination with antibiotics, phages or phages + antibiotics was monitored via 600nm absorbance every 20 min after 5 second linear shaking. Measurements were performed in triplicates and plotted as mean ± SD (to improve readability error bars were plotted for every tenth data point).

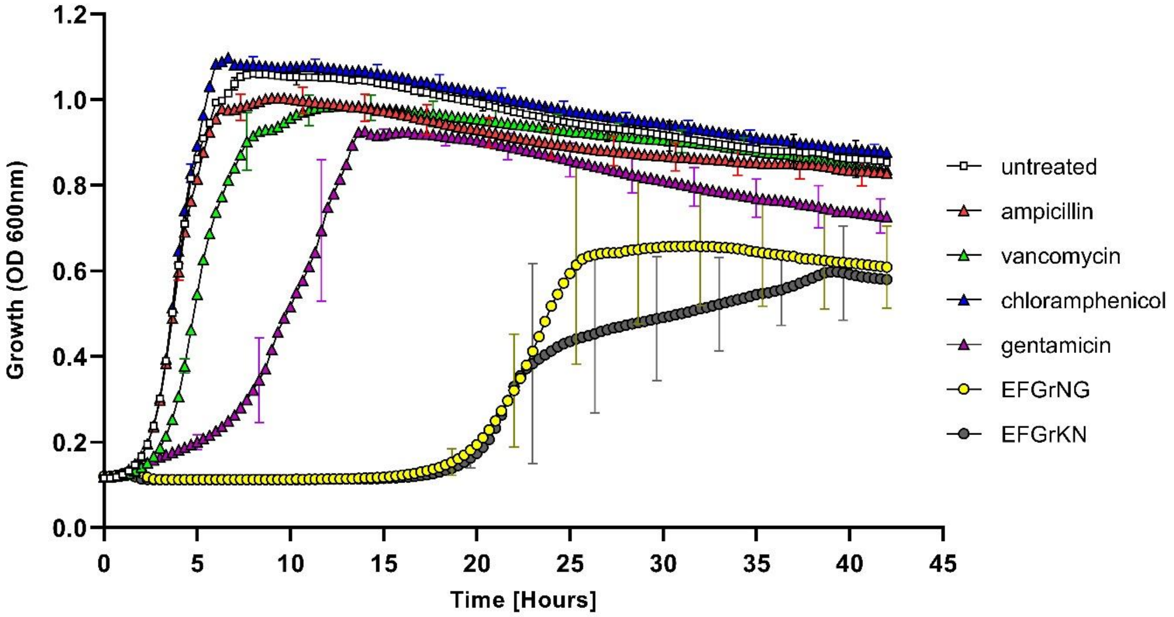

Figure S1. The inhibition effects of phages (circles) and antibiotics (triangles) on the target bacteria VREfm (squares).

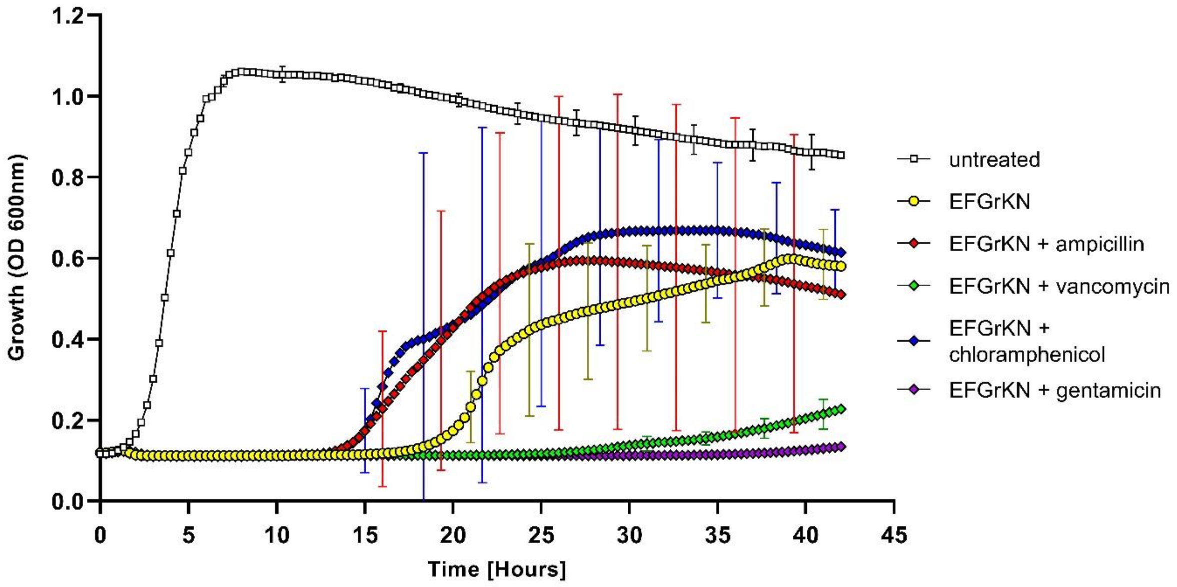

Figure S2. Combined effect of phage EFGrKN and antibiotics on the target bacteria VREfm

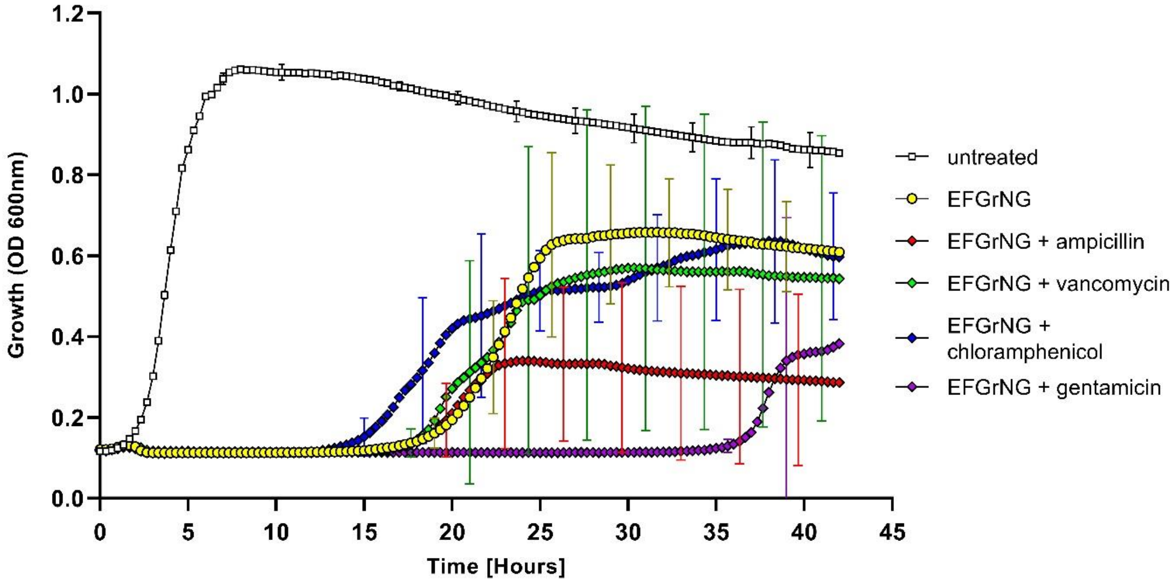

Figure S3. Combined effect of phage EFGrNG and antibiotics on the target bacteria VREfm.
